# Access to Specialist Care in Rural Saskatchewan: The Saskatchewan Rural Health Study

**DOI:** 10.3390/healthcare3010084

**Published:** 2015-02-16

**Authors:** Chandima P. Karunanayake, Donna C. Rennie, Louise Hagel, Joshua Lawson, Bonnie Janzen, William Pickett, James A. Dosman, Punam Pahwa, The Saskatchewan Rural Health Study Group

**Affiliations:** 1 Canadian Centre for Health and Safety in Agriculture, University of Saskatchewan, 104 Clinic Place, Saskatoon, SK S7N 2Z4, Canada; E-Mails: donna.rennie@usask.ca (D.C.R.); louise.hagel@usask.ca (L.H.); josh.lawson@usask.ca (J.L.); james.dosman@usask.ca (J.A.D.); pup165@mail.usask.ca (P.P.); 2 College of Nursing, University of Saskatchewan, 104 Clinic Place, Saskatoon, SK S7N 2Z4, Canada; 3 Department of Medicine, University of Saskatchewan, Royal University Hospital, 103 Hospital Drive, Saskatoon, SK S7N 0W8, Canada; 4 Department of Community Health & Epidemiology, College of Medicine, University of Saskatchewan, 107 Wiggins Road, Saskatoon, SK S7N 5E5, Canada; E-Mail: bonnie.janzen@usask.ca; 5 Department of Public Health Sciences, Queens University, Kingston, ON K7L 3N6, Canada; E-Mail: will.pickett@queensu.ca

**Keywords:** rural, specialist care, distance

## Abstract

The role of place has emerged as an important factor in determining people’s health experiences. Rural populations experience an excess in mortality and morbidity compared to those in urban settings. One of the factors thought to contribute to this rural-urban health disparity is access to healthcare. The objective of this analysis was to examine access to specialized medical care services and several possible determinants of access to services in a distinctly rural population in Canada. In winter 2010, we conducted a baseline mail survey of 11,982 households located in rural Saskatchewan, Canada. We obtained 4620 completed household surveys. A key informant for each household responded to questions about access to medical specialists and the exact distance traveled to these services. Correlates of interest included the location of the residence within the province and within each household, socioeconomic status, household smoking status, median age of household residents, number of non-respiratory chronic conditions and number of current respiratory conditions. Analyses were conducted using log binomial regression for the outcome of interest. The overall response rate was 52%. Of households who required a visit to a medical specialist in the past 12 months, 23% reported having difficulty accessing specialist care. The magnitude of risk for encountering difficulty accessing medical specialist care services increased with the greatest distance categories. Accessing specialist care professionals by rural residents was particularly difficult for persons with current respiratory conditions.

## 1. Introduction

Access to medical and other healthcare services is a fundamental determinant of health outcomes [1]. Access to healthcare varies across countries, groups and individuals, largely influenced by social and economic conditions, as well as the health policies in place. Canada’s healthcare system is governed by the Canada Health Act (CHA), which ensures that primary care doctors, specialists, hospitals and dental surgeries are covered through provincial health insurance plans [2,3]. The CHA is Canada’s federal legislation for publicly-funded healthcare insurance. This is designed to ensure that all residents have reasonable access to medically necessary hospital and physician services on a prepaid basis. Instead of having a single national plan, Canada’s national health insurance program is composed of 13 provincial and territorial health insurance plans, all of which share certain common features and basic standards of coverage. Primary healthcare is the foundation of the healthcare system and serves a dual function. It is the first point of contact with the healthcare system, and primary care providers act as gatekeepers for further care to ensure continuity of care and ease of movement across the healthcare system when more specialist services are needed [2]. In many commonwealth countries, such as Australia, England, Italy, New Zealand and Denmark, primary healthcare providers act as gatekeepers for further care similar to Canada. However, how the governments fund and manage the healthcare system varies by country [4].

Approximately 19% of Canada’s total population lives in rural areas [5]. Health status observed among rural Canadians is generally lower than that of their urban counterparts [6]. In the general Canadian population, the majority of individuals who accessed a medical specialized service did not experience any difficulties; however, some did. Approximately 11% of those 15 years of age or older visited a medical specialist in 2005, and among them, 19% reported that they faced difficulties accessing specialist care [7]. Despite the fact that the Canada Health Act includes a guarantee of reasonable access to healthcare services for all Canadians, rural residents have access to a much narrower range of services than their urban counterparts [8]. Physicians are not evenly distributed throughout Canada, and many rural Canadians have more limited access to healthcare providers, rural hospitals and other health services. In 1996, 9.8% of Canada’s physicians practised in rural and remote areas compared with 14.9% in 1991 [6]. Access to healthcare is also a problem, not only because these communities struggle to attract and keep nurses, doctors and other healthcare providers, but also because of distances [9,10]. Canada has one of the largest land masses in the world, and Canadians are often required to travel long distances to receive healthcare. Costs due to travel are also a challenging reality for rural patients [9]. The waiting period is another major problem in access to specialist care. In 2009, about 46.4% of Canadians aged 15 and older reported waiting over a month for a specialist physician visit, with 14.4% waiting more than three months [7]. There were 11.6 weeks of actual median wait time between referral by a general physician and appointment with a specialist in Saskatchewan in 2013, compared to the actual national median of 8.6 weeks [11]. A similar trend (14.1 weeks *vs.* 9.6 weeks) was observed for actual wait time for appointment with a specialist and treatment in 2013 [11]. In a comparison of the wait times for a specialist appointment in 11 commonwealth countries, Canadians reported the longest wait for a specialist appointment: 41% of Canadian residents waited two months or more compared to only 5%, 7% and 9% of Swiss, German and American residents, respectively [4].

Very little evidence exists about rural Canadian populations and their access to specialist care services. The Saskatchewan Rural Health Study (SRHS) [12] is a large population health study aimed, in part, at addressing some of these gaps in knowledge in rural Saskatchewan. The objective of this analysis was to evaluate the nature and extent of access to medical specialist care services in rural populations and to examine relations between distances to medical specialist care services and difficulty accessing specialist care services for persons with respiratory health outcomes in rural Saskatchewan.

## 2. Methods

### 2.1. Baseline Survey

**Figure 1:**
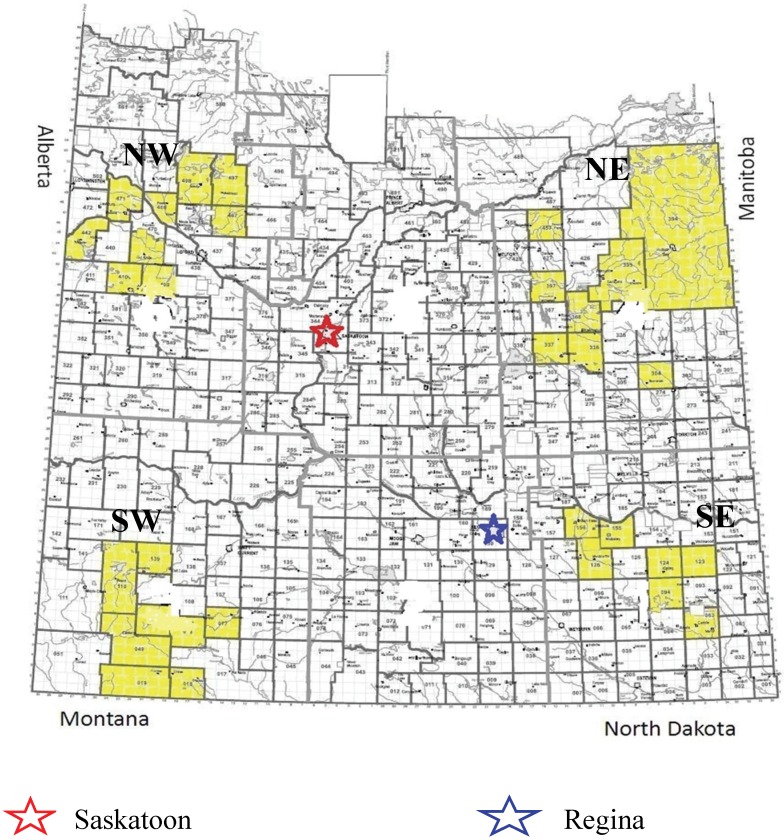
Map of study quadrants in the province of Saskatchewan, Canada.

The SRHS design is a prospective rural cohort study being conducted in two phases, baseline and follow-up. Details of the study design for the baseline survey have been reported elsewhere [12]. The study was approved by the Biomedical Research Ethics Board of University of Saskatchewan, Canada (Bio#09-56; approved on 9 April 2009). The rural population is defined as consisting of those persons living in towns and municipalities outside the commuting zone of larger urban centres with a population of 10,000 or more [13]. In 2006, there were 44,329 farm enterprises in Saskatchewan encompassing 111,600 household residents [14]. In brief, 39 rural municipalities (RMs) of the 298 RMs in Saskatchewan and 16 of the 145 towns (population ranging from 500 to 5000) in Saskatchewan were selected to participate in the study. These RMs and towns were selected at random from four quadrants of the province (southwest (SW), southeast (SE), northeast (NE) and northwest (NW); see Figure 1). The local councils for most of these communities, 32 out of 39 RMs and 15 out of 16 towns, agreed to participate on behalf of their residents and supplied mailing addresses. Dillman’s method was utilized to recruit study participants [15]. The resultant study population was comprised of 8261 individuals (males and females, 18 years of age or older) from 4624 households (4620 completed questionnaires). Information was collected by self-administered, mailed questionnaires based on the Population Health Framework [16,17].

**Figure 2:**
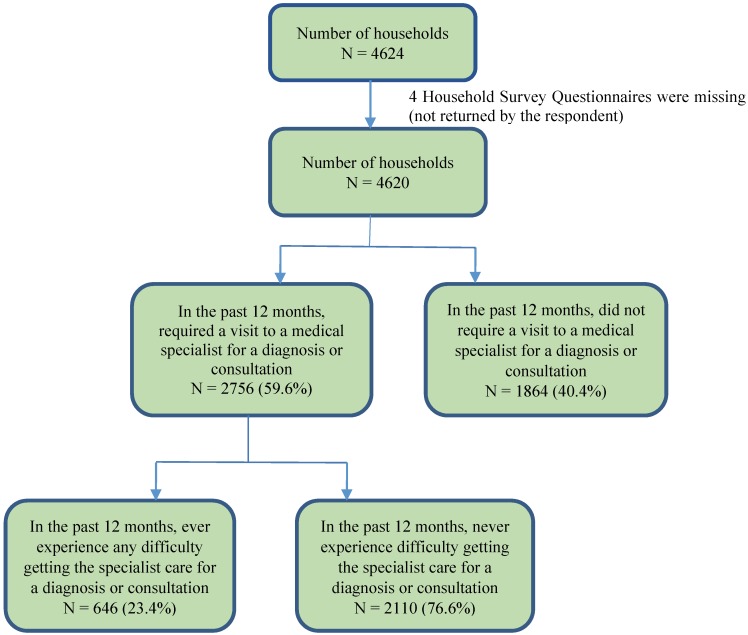
Sampling frame of access to specialist care in the past 12 months.

Several measures of lifestyle factors, occupational exposures and socio-economic status used in our questionnaire were adopted from previous research studies that had validated these measures [16,18,19]. The survey consists of two questionnaires that supplied individual and household information, respectively. Details of access to healthcare were obtained at the household level. Analyses in this study were restricted to the 2756 households indicating having required a visit to a medical or surgical specialist for a diagnosis or consultation for themselves or a family member in their household in the past 12 months (Figure 2).

### 2.2. Study Variables

The main study outcome derived from the household questionnaire was: “in the past 12 months, have you ever experienced any difficulties getting the medical or surgical specialist care you needed for a diagnosis or consultation for yourself or a family member in your household?”

Difficulties in accessing medical or surgical specialist care services were assessed by the distance required to travel to receive these medical or surgical specialist services. Distance to specialist care was grouped into tertiles: (0–120 km, >120–200 km and >200 km). This categorization addresses the effect of the right tail skewness of the distribution. Potential confounders included: (i) designation of residence in a rural dwelling as farm or non-farm (acreage, or in town); (ii) the four quadrants of the province (SW, SE, NE and NW; see Figure 1); (iii) metropolitan influence zones (MIZ) defined as no MIZ (a census subdivision (CSD), where there are fewer than 40 persons or none commute to work in an urban core), weak MIZ (a CSD where more than 0%, but less than 5% of residents commute to work in an urban core and excluding CSDs with fewer than 40 persons in their resident employed labour force) or moderate MIZ (a CSD where at least 5%, but less than 30% of residents commute to work in an urban core and excluding CSDs with fewer than 40 persons in their resident employed labour force); all of these three zones have population sizes of <10,000 [20]; (iv) socioeconomic status was assessed using household income adequacy, which was a derived variable with four categories based on various combinations of total household income and the number of people living in the household, according to a Statistics Canada definition [21]; (v) household smoking was collected by the following question: “Do any of the people who live in your house use any of the following tobacco products in the home? (cigarettes: yes/no/don’t know; cigars: yes/no/don’t know; pipes: yes/no/don’t know)”; (vi) median age of the household; (vii) total number of non-respiratory chronic conditions (ever) in the household, such as diabetes, heart disease, heart attack, hardening of the arteries, high blood pressure, cystic fibrosis, tuberculosis, stroke and cancer; and (viii) total number of current respiratory conditions reported for the past 12 months (asthma, hay fever, sinus trouble, chronic bronchitis, emphysema, chronic obstructive pulmonary disease and sleep apnea). The total number of non-respiratory chronic conditions and the total number of current respiratory conditions were then categorized into three groups: none, one condition and two or more conditions.

### 2.3. Missing Data

Missing data were evident for several key variables. However, for most variables, there was no difference in results when analyses with and without missing values were conducted. Responses were not provided for 417 (15.1%) of the population for the variable “household income adequacy”. Simply dropping records with missing data from the analysis could lead to misleading conclusions, because the complete records may be unrepresentative of the population of interest. Therefore, a new category, called “not stated/unknown” was included in the analysis in the household income adequacy variable.

Of the 2756 respondents who reported one or more visits to medical specialist for a diagnosis or medical consultation for a household member, 202 (7.3%) did not complete the distance to specialist services item. To eliminate the potential bias due to missing responses, missing values were replaced according to the MIZ in which they lived using three distance categories: no MIZ (>200 km), weak MIZ (>120–200 km); moderate MIZ (0 to 120 km).

### 2.4. Statistical Analysis

Statistical analyses were completed using SAS Version 9.3 (SAS Institute, Cary, NC, USA). Prevalences were presented as observed/total and percentages. In this study, log-binomial regression models [22,23] with the log function were used to compute adjusted relative risk estimates and associated 95% confidence intervals that summarized relations between difficulty accessing medical specialist care services (yes or no) and distance to the medical specialist care services and other explanatory variables. In general, logistic regression yields an odds ratio that approximates the relative risk when the event is rare, adjusting for potential confounders. For more common outcomes, the odds ratio may overestimate the relative risk. In this situation, the log-binomial regression [22,23] has been proposed to compute a less biased adjusted relative risk. The sample size of 4620 households resulted in adequate power to detect a relative risk of 1.25 at an α level of 0.05 with 2-sided tests of significance.

## 3. Results

The overall response rate at the household level was 52%. Of the 2,756 households that required a visit to a medical specialist for a diagnosis or consultation in the past 12 months, almost everyone (99.8%) had access to a regular doctor or nurse practitioner. Of the 2756 households requiring a visit to a medical specialist for diagnosis or consultation in the past 12 months, 646 (23.4%) reported having difficulty obtaining services. About one third (32.5%) of the respondents reported that they were travelling more than 200 km to visit a medical specialist for diagnosis or consultation (Table 1).

Results of the log binomial logistic regression analysis are reported in Table 2. The relative risk for difficulty accessing medical special care was 1.12 (0.93–1.34) and 1.45 (1.21–1.74) for distances >120–200 km and >200 km, respectively. Difficulty in accessing services was greater for farm than non-farm households (relative risk (95% CI): 1.19 (1.04–1.37)). There was also greater difficulty in obtaining medical specialist consultation care for households located in the southwest (relative risk (95% CI): 1.46 (1.19–1.79)) and southeast (relative risk (95% CI): 1.34 (1.09–1.64)) regions of the province compared to the northwest. Difficulties in access were higher for those reporting current respiratory conditions in the household (Table 2). We did not observe any association between access to medical specialist care and MIZ, household income adequacy, median age of the household, household smoking status and number of non-respiratory chronic conditions.

**Table 1:** Frequency of access and distance to healthcare services within the Saskatchewan Rural Health Study (SRHS) households.

Variable	*n*	%
In the past 12 months, required a visit to a medical specialist for a diagnosis or consultation (*N* = 4620)		
Yes	2756	59.6
No	1864	40.4
Have access to regular doctor or nurse practitioner (of those who access the services; *N* = 2756)		
Yes	2752	99.8
No	4	0.2
In past 12 months, ever experienced any difficulty getting the specialist care for a diagnosis or consultation (of those who access the services; *N* = 2756)		
Yes	646	23.4
No	2110	76.6
Distance to medical specialist care in the past 12 months for the households requiring a visit to a medical specialist for a diagnosis or consultation (of those who access the services (*N* = 2756); missing: *n* = 202)		
0–120 km	920	36.0
>120–200 km	806	31.6
>200 km	828	32.4
Distance to medical specialist care in the past 12 months for the households requiring a visit to a medical specialist for a diagnosis or consultation (of those who access the services (*N* = 2756); after replacing missing values)		
0–120 km	952	34.5
>120–200 km	907	32.9
>200 km	897	32.5

**Table 2:** Results of log binominal regression analysis examining associations between access to specialist healthcare in the past 12 months and distance.

Household Characteristics	In the Past 12 Months, Experienced Difficulties Getting Medical Specialist Care for a Diagnosis or Consultation *n* = 2756				
	n/Total	%	Crude Relative Risk (95% CI)	Adjusted Relative Risk (95% CI)	Adjusted Relative Risk after replacing missing distances (95% CI)
**Home Location**					
Farm	267/1037	25.8	**1.17 (1.02, 1.34)**	**1.16 (1.01, 1.34)**	**1.19 (1.04, 1.37)**
Non-Farm	376/1703	22.1	1.00	1.00	1.00
**Quadrant**					
Southwest	157/503	31.2	**1.59 (1.32, 1.92)**	**1.49 (1.21, 1.83)**	**1.46 (1.19, 1.79)**
Southeast	158/604	26.2	**1.34 (1.10, 1.62)**	**1.39 (1.13, 1.71)**	**1.34 (1.09, 1.64)**
Northeast	162/785	20.6	1.05 (0.87, 1.28)	0.99 (0.78, 1.26)	0.93 (0.74, 1.17)
Northwest	169/863	19.6	1.00	1.00	1.00
**Metropolitan Influence Zone (MIZ)**					
No MIZ	187/766	24.4	1.10 (0.89, 1.37)	0.93 (0.73, 1.19)	0.96 (0.75, 1.22)
Weak MIZ	359/1537	23.4	1.06 (0.87, 1.28)	0.93 (0.75, 1.15)	0.95 (0.77, 1.18)
Moderate MIZ	100/452	22.1	1.00	1.00	1.00
**Household Income Adequacy**							
Not stated/unknown	93/417	22.3	0.85 (0.69, 1.05)	0.94 (0.76, 1.16)	0.94 (0.76, 1.16)
Lowest	35/130	26.9	1.03 (0.76, 1.39)	1.19 (0.87, 1.64)	1.22 (0.90, 1.65)
Lowest middle	101/462	21.9	0.84 (0.68, 1.02)	0.90 (0.72, 1.12)	0.96 (0.78, 1.19)
Upper middle	166/787	21.1	**0.81 (0.67, 0.96)**	0.85 (0.72, 1.01)	0.87 (0.73, 1.03)
Highest	251/960	26.2	1.00	1.00	1.00
**Household Smoking**					
Yes	92/413	22.3	0.94 (0.77, 1.14)	0.87 (0.71, 1.06)	0.90 (0.74, 1.09)
No	552/2329	23.7	1.00	1.00	1.00
**Median Age**					
>65 years	179/921	19.4	**0.76 (0.62, 0.92)**	0.84 (0.67, 1.06)	0.85 (0.68, 1.07)
>55–65 years	154/636	24.2	0.94 (0.78, 1.15)	1.01 (0.81, 1.25)	1.01 (0.82, 1.25)
>45–55 years	169/639	26.5	1.03 (0.85, 1.25)	1.04 (0.85, 1.27)	1.04 (0.86, 1.27)
18–45 years	143/558	25.6	1.00	1.00	1.00
**Non-Respiratory chronic conditions**					
2 or more conditions	221/1018	21.7	0.86 (0.74, 1.02)	0.89 (0.74, 1.08)	0.90 (0.74, 1.08)
One condition	192/809	23.7	0.95 (0.80, 1.12)	0.98 (0.82, 1.17)	0.94 (0.79, 1.12)
None	233/929	25.1	1.00	1.00	1.00
**Current Respiratory conditions**					
2 or more conditions	184/638	28.8	**1.49 (1.26, 1.75)**	**1.37 (1.16, 1.61)**	**1.39 (1.18, 1.63)**
One condition	198/758	26.1	**1.35 (1.14, 1.58)**	**1.24 (1.05, 1.47)**	**1.29 (1.09, 1.51)**
None	264/1360	19.4	1.00	1.00	1.00
**Distance to medical specialist care**					
>200 km	235/828	28.4	**1.39 (1.18, 1.65)**	**1.45 (1.21, 1.73)**	**1.45 (1.21, 1.74)**
>120–200 km	190/806	23.6	1.16 (0.97, 1.39)	1.13 (0.94, 1.36)	1.12 (0.93, 1.34)
0–120 km	187/920	20.3	1.00	1.00	1.00

## 4. Discussion

Limited access to specialist care remains a major barrier to healthcare in Canada, and wait times appear to be increasing [24]. In 2009, 14.4% of adults in the general Canadian population waited longer than three months for specialist physician visit, compared to 10.4% in 2003 [7]. Structural changes to Canada’s healthcare system in how wait times are mitigated, measured, monitored and managed are long overdue [25]. Another reason for increasing wait times is the continuing shortage of specialists due to aging and retirement of the medical work force, especially surgical and medical specialists [26]. In this study of rural Canadians, nearly one-quarter of those requiring specialist care reported challenges for accessing that care. The risk of encountering difficulty in accessing medical specialist care services increased with greater distances. Accessing specialist care professionals by rural residents was particularly difficult for persons with current respiratory conditions. In addition, farm households in this study reported more difficulty in accessing services than small town (non-farm) households. Farms in Western Canada can be located several kilometers from local health services, as shown in the survey, which could influence travel decisions to access care. Furthermore, the quality of rural roads and certain seasons may interfere with timely access to care. Participants from the southwest and southeast regions of the province were more likely to experience difficulty accessing specialist care services compared to those in the northwest.

Distances to regional healthcare centers can often be great, especially in the most rural areas of Canada. A number of studies have measured the impact of distance on healthcare utilization and have reported findings similar to ours [27]. Distance to regular care services was found to have a significant negative relationship with the number of regular care check-up visits in a study of rural North Carolina [28]. Nemet and Bailey [29] studied the association between distance and utilization of healthcare by a group of elderly residents in rural Vermont, confirming that increased distance from healthcare providers reduced utilization of healthcare. Winters *et al.* [30] conducted a study on the self-management of chronic illness by women living in isolated, rural areas of five western U.S. states. Distance had a significant impact in that context, as well.

Respiratory diseases, excluding lung cancer, represent the fourth leading burden to healthcare in Canada [31]. Guidelines provided by the Canadian Thoracic Society for the management of chronic obstructive pulmonary disease (COPD) [32], asthma [33], sleep disordered breathing [34], tuberculosis (TB) [35] and the American College of Chest Physicians (ACCP) Evidence-Based Clinical Practice Guidelines for diagnosis and management of cough [36] report that referral to a specialist is appropriate in certain circumstances. COPD patients are referred to a specialist in the face of diagnostic uncertainty, when symptoms are disproportional to the level of airflow obstruction, when there is accelerated decline of pulmonary function, onset of symptoms at a young age or when there is severe or recurrent COPD and failure to respond to therapy [32]. Asthma patients are referred to a specialist when their condition remains uncontrolled after treatment of inhaled corticosteroids, when there is a need to administer higher doses of inhaled corticosteroids and when there is the use of oral corticosteroids for an episode of acute loss of asthma control for all age groups [33]. Patients should be referred for medical specialist assessment when they are suspected cases of sleep disordered breathing, with major daytime sleepiness (Epworth Sleepiness Score (ESS) of 15 or greater), and when they have comorbid conditions, with and without a safety-critical occupation [34]. When there is uncertainty and several medications to administer, consultation with a tuberculosis specialist is recommended [35]. According to ACCP guidelines, patients with a high suspicion of cough due to environmental or occupational exposures and patients with uncontrolled cough are to be referred to a cough specialist [36].

Patients diagnosed with respiratory diseases, such as asthma and COPD, are primarily cared for by primary care practitioners. If specialist care is needed, referral to a respirologist/pulmonologist, allergist or pediatric respirologist is made [31]. Patients diagnosed with conditions, like tuberculosis, cystic fibrosis and lung cancer, require treatment by medical specialists [31]. Past research suggests that patients with respiratory conditions, who were referred to a consulting specialist often described that the waiting time was too long [31]. Another study looked at the impact of living in a rural area with advanced chronic respiratory illness and found that the distance was a significant challenge to accessing healthcare [37]. Our findings were similar in that they indicated that the risk of experiencing difficulty of getting medical specialist consultation was higher for participants with any current respiratory conditions compared to no respiratory conditions.

The cost of physician services is not a barrier for any Canadian’s ability to see a doctor. However, the distance to be traveled may restrict some people’s access to health services [38]. The distance traveled to physician services can be an indirect economic cost related to healthcare, particularly for those who live in more remote areas. A Canadian study exploring barriers to predictive testing for Huntington’s disease in British Columbia revealed that the accessibility of specialists can be a barrier for this group of patients for two major reasons: (1) travel distances; and (2) inflexibility of the testing process [39]. The Romanow Commission [40] on the Future of Health Care in Canada was a federal public inquiry created in April, 2001, to review and make recommendations regarding Canada’s public healthcare system. The Romanow Commission objectives were to inquire into, and gather information regarding, the future of Canada’s public healthcare system and to make recommendations to government in regards to the public health system’s long-term stability. According to the Romanow Report, “People in rural and remote communities have poorer health status than Canadians who live in larger centres. Access to health care is also a problem, not only because of distances, but because these communities struggle to attract and keep nurses, doctors and other health care providers” [40].

A study reported from Canadian Community Health Survey (CCHS 2.1) conducted in 2003 reported that the number of chronic conditions (respiratory and non-respiratory) was a very strong predictor of access to healthcare [41]. The same study reported that among the rural categories, there was a gradient with the most rural (no or weak metropolitan influence zone (MIZ)) being least likely to have had a flu shot, use specialist physicians services or have a regular medical doctor. A recent study based on 2010 CCHS data for the province of Ontario, Canada [3], revealed that the number of chronic conditions and health region type (e.g., urban/rural) were associated with an increased likelihood of experiencing difficulties accessing specialist care in the previous year. Further, respondents with 1–3 chronic conditions were 2.2-times as likely to experience difficulties compared to those with no conditions, and those with four or more chronic conditions were almost four-times as likely to have had difficulties. Compared to respondents from the city of Toronto, those from other urban health regions and rural regions were more likely to report difficulties accessing specialist care. Similar to these national and provincial studies, we also observed that current respiratory conditions were associated with a higher odds of experiencing difficulties accessing specialist care. We did not observe a relationship between household income and access to specialist care in our study. In contrast to our findings, Sibley *et al.* [41] did find an association between household income and access to medical care, with those in the lowest household income quartile being less likely to have had a flu shot, to have seen a specialist or to have a regular medical doctor. Although other research has similarly reported a relationship between lower household income and greater difficulty accessing specialist/physician care [42,43], no other studies, to our knowledge, have examined whether this association holds true in rural environments. Further research is needed to explore the relationship between household income adequacy and access to specialist care in rural settings.

Access to specialist care can be improved through the use of different techniques, such as teleconsultation. Communication with a specialist can be done using a secure web-based system. E-consultation has been suggested to improve access to specialist care recently [44]. The e-consultation system allows primary care providers to submit patient’s information to a specialist, using a standardized web-based form. In 2014, there were over 235 telehealth sites in Saskatchewan in 100 communities across the province, and over 7500 patients utilized telehealth services for their clinical consultation [45]. By using these services, access to specialist care can be improved and wait times reduced, and this is associated with a reduction in the number of hospital visits and result in savings to patients in terms of costs and time [44,46]. Although the web-based system affords opportunities for rural Canadians to reduce the barriers of distance, Internet use is lower in rural Canada [47]. In 2012, 85% of rural households had access to broadband, compared to 100% of urban households [48].

The main strength of this study is that it is based on a very large and representative sample of the rural population. Another strength is that the place of residence was examined from several perspectives, including: farm/non-farm residence, MIZ and quadrant level. The limitations of this study are largely related to the design of the SRHS. Because the study is based on survey data, there is a risk of misclassification of the variables under study. Respondents were requested to recall their access to specialist care in the past 12 months. Furthermore, the lack of detailed clinical data limited the ability to adjust for chronic condition severity in regression models. Another limitation is that 95% of the households were of Caucasian origin and did not include First Nations populations living on rural reserves. This may result in overestimating the level of access in rural areas. About 50% of Canada’s aboriginal population lives in rural and remote areas, and they were less likely to access more specialized healthcare due to the low retention of health professionals and the lack of transportation infrastructure [49].

## 5. Conclusions

The purpose of the present study was to examine access to specialized medical care services and possible determinants in a distinctly rural population in Canada. The results reveal that accessing specialist care professionals by rural residents was particularly difficult for persons with current respiratory conditions. Furthermore, the magnitude of the risk for encountering difficulty accessing medical specialist care services increased with the greatest distance categories. Attempts to reduce barriers to accessing specialist care should include measures to reduce the distance people are required to travel to specialist healthcare-related services.
